# Supplementary material for: CCR5 deficiency impairs CD4+ T‐cell memory responses and antigenic sensitivity through increased ceramide synthesis
Source: EMBO J. 2020 Jun 11;39(15):e104749. doi: 10.15252/embj.2020104749 (PMC7396835; doi:10.15252/embj.2020104749)
Supplement: Supplementary file 1 — Appendix [file EMBJ-39-e104749-s001.pdf]

CCR5 deficiency impairs CD4^{+} T cell memory responses and antigenic sensitivity through increased ceramide synthesis

Ana Martín-Leal, Raquel Blanco, Josefina Casas, María E. Sáez, Elena Rodríguez-Bovolenta, Itziar de Rojas, Carina Drechsler, Luis Miguel Real, Gemma Fabrias, Agustín Ruíz, Mario Castro, Wolfgang W.A. Schamel, Balbino Alarcón, Hisse M. van Santen, Santos Mañes

Supplemental information

Appendix Figure S1 – S8

Appendix Tables S1 – S3

Bayesian code

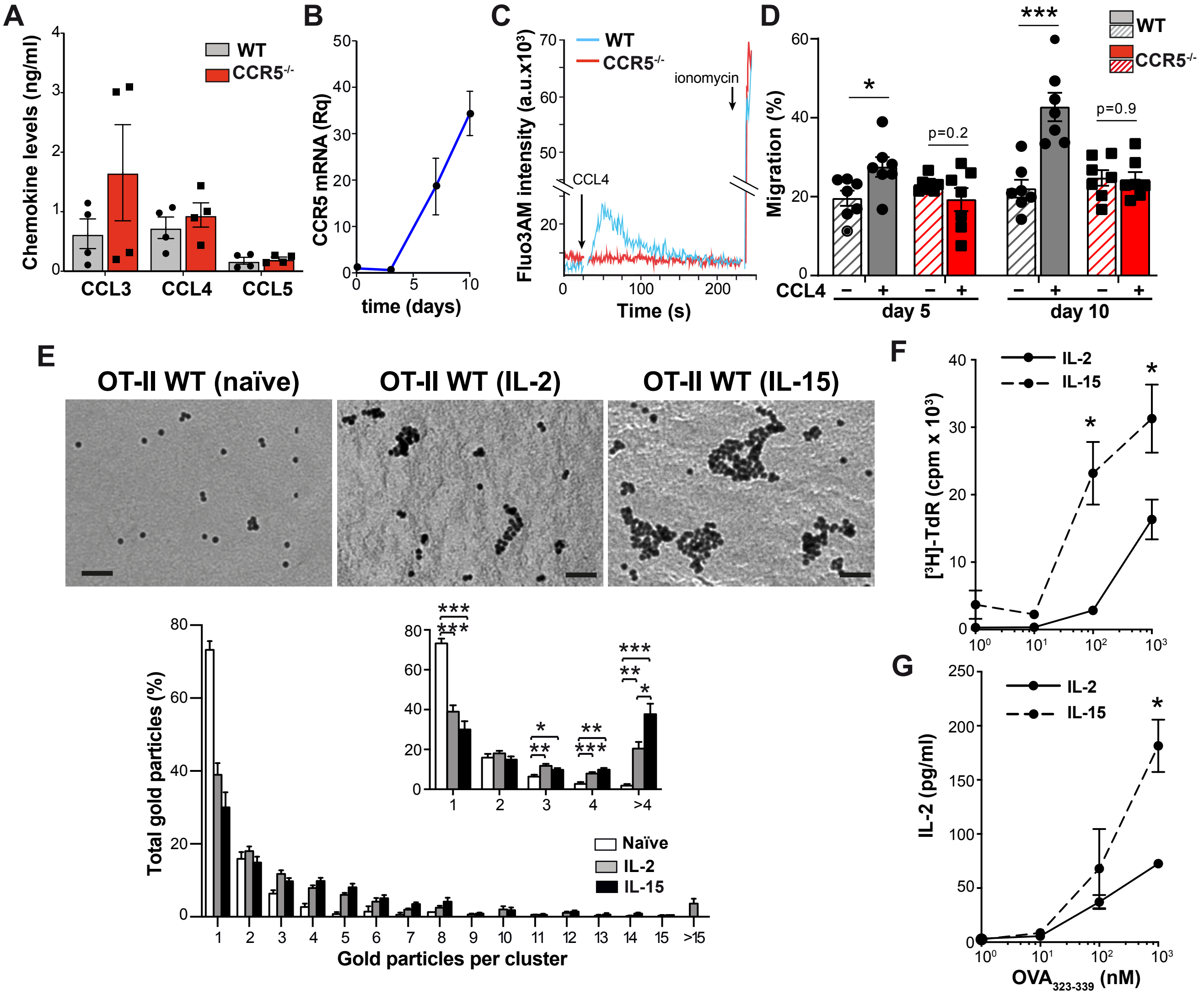

Appendix Figure S1. Characterization of CD4^{+} T lymphoblasts. A. CCR5 ligand levels in supernatants of day 10 IL-2-expanded OT-II WT and CCR5$^{-/-}$ lymphoblasts. Data shown as mean ± SEM (n = 4). **B.** Relative CCR5 mRNA levels in OT-II WT lymphoblasts. mRNA was not detected in OT-II CCR5$^{-/-}$ lymphoblasts. **C.** Time course of intracellular Ca^{2+} mobilization in response to CCL4 in OT-II WT and CCR5$^{-/-}$ lymphoblasts. Cells were finally treated with ionomycin as a positive control of Ca^{2+} loading (n = 3). **D.** Analysis of OT-II WT and CCR5$^{-/-}$ lymphoblast transmigration in transwell chambers, using CCL4 as chemoattractant (solid bars) or in basal medium (dashed bars). Data are the percentage of migrating cells relative to total cells seeded. **E.** EM analysis of TCR nanoclustering in OT-II WT naïve cells and lymphoblasts expanded in IL-2 or IL-15. Top, representative small field images showing gold particle distribution in the cell surface replicas of anti-CD3ε-labeled cells. Bottom, quantification (mean ± SEM) of gold particles in clusters of the indicated size in the IL-2- (gray bars; n = 6 cells, 27518 particles) and IL-15-expanded lymphoblasts (black; n = 8 cells, 27518 particles). Insets show the distribution between clusters of one, two, three, four or more than four particles, and statistical analysis. **F, G.** IL-2- and IL-15-expanded OT-II WT lymphoblasts were restimulated with the indicated concentrations of $OVA_{323\text{-}339}$; cell proliferation measured by thymidine incorporation into DNA (*F*) and IL-2 production measured by ELISA (*G*) were determined after 72 h of stimulation. Data shown as mean ± SEM (n = 5). * p <0.05, ** p <001, *** p <0.001, two-tailed unpaired Student's *t*-test. Bar, 50 nm.

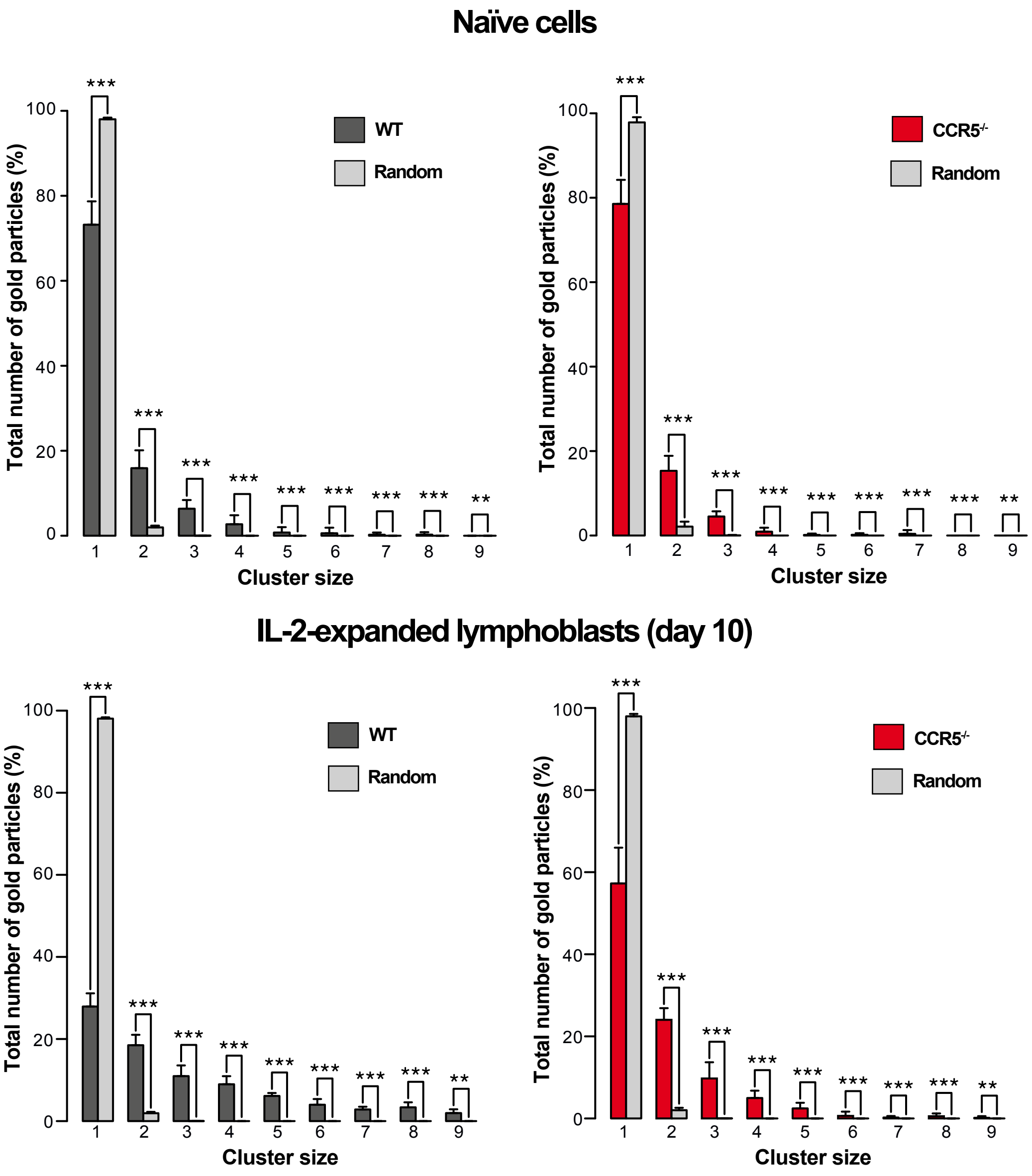

Appendix Figure S2. Comparison of experimental and synthetic TCR multimer distributions. Percentage of clusters of size *n* (1 to 9) in WT and CCR5$^{-/-}$ OT-II naïve cells and lymphoblasts (day 10) determined experimentally (dark bars), and synthetically random generated receptors (light bars). Student's *t*-test significance for each cluster size is shown above bars (p >0.05 (not significant), * p <0.05, **p <0.01, *** p <0.001). In all cases, the experimental distributions of clusters differ significantly from random proximity between clusters.

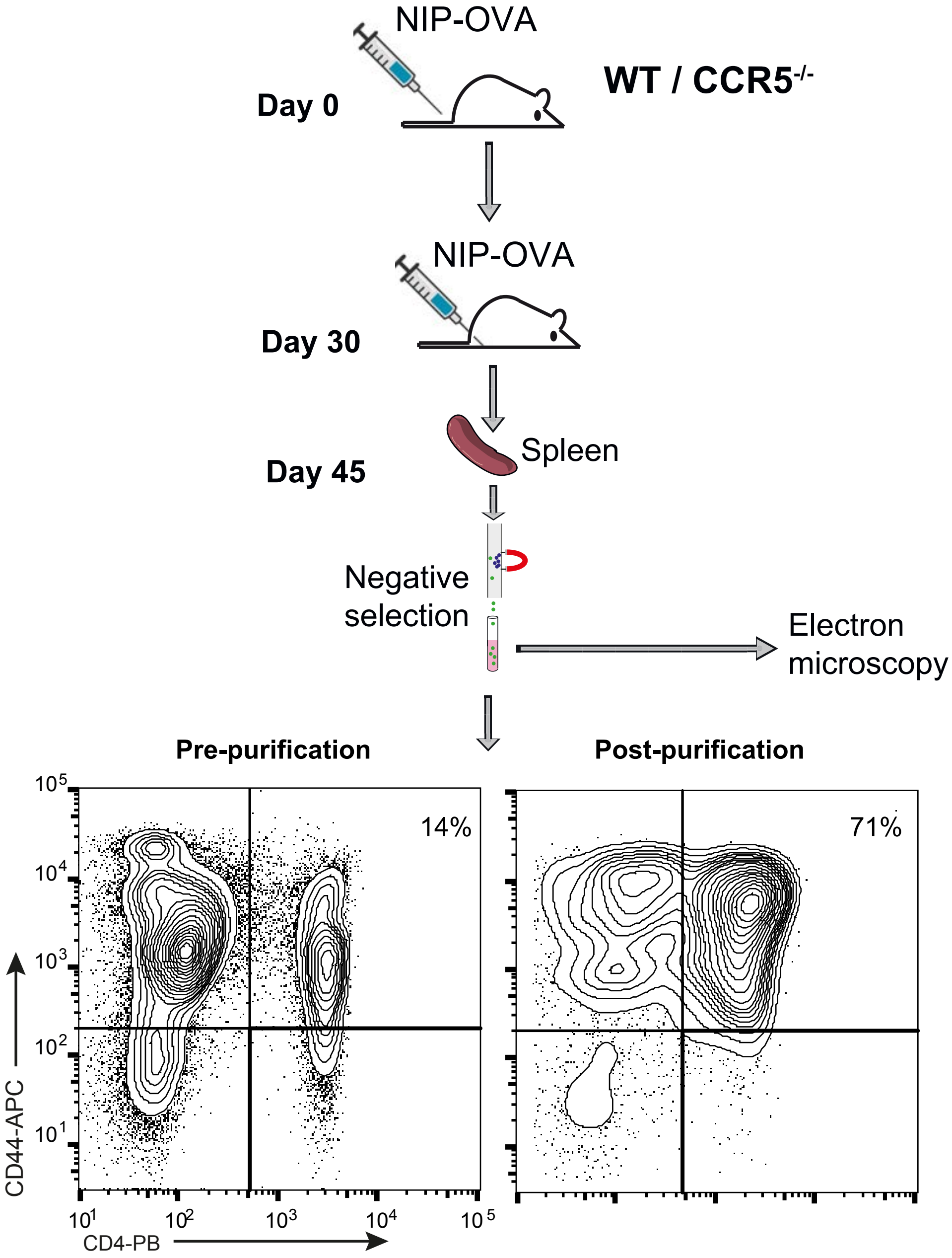

Appendix Figure S3. Characterization of endogenous memory CD4⁺ T cells uses for electron microscopy studies. Scheme of the purification of memory cells from OVA/OVA-immunized mice as well as a representative plot showing the characterization of the purified cells by flow cytometry.

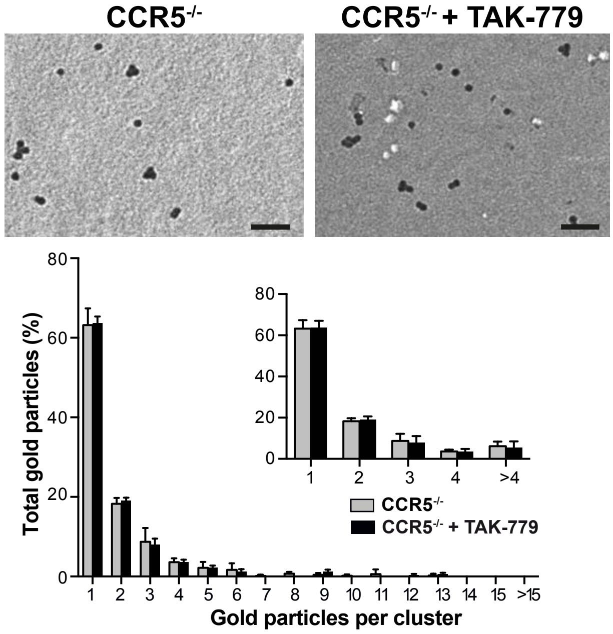

Appendix Figure S4. TAK-779 does not affect TCR nanoclustering in CCR5$^{-/-}$ lymphoblasts. OT-II CCR5$^{-/-}$ cells were activated with OVA$_{323-339}$ for 3 days in the presence of TAK-779, and lymphoblasts were generated by expansion with IL-2. Top, representative small field images showing gold particle distribution in cell surface replicas of anti-CD3ε-labeled cells. Bottom, quantification (mean ± SEM) of gold particles in clusters of the indicated size in vehicle-treated (gray bars; n = 6 cells, 5138 particles) and TAK-779-treated lymphoblasts (black; n = 5 cells, 4215 particles). Insets, distribution between clusters of one, two, three, four or more than four particles, and statistical analysis. * $p < 0.05$, one-tailed unpaired Student's t-test. Bar, 50 nm.

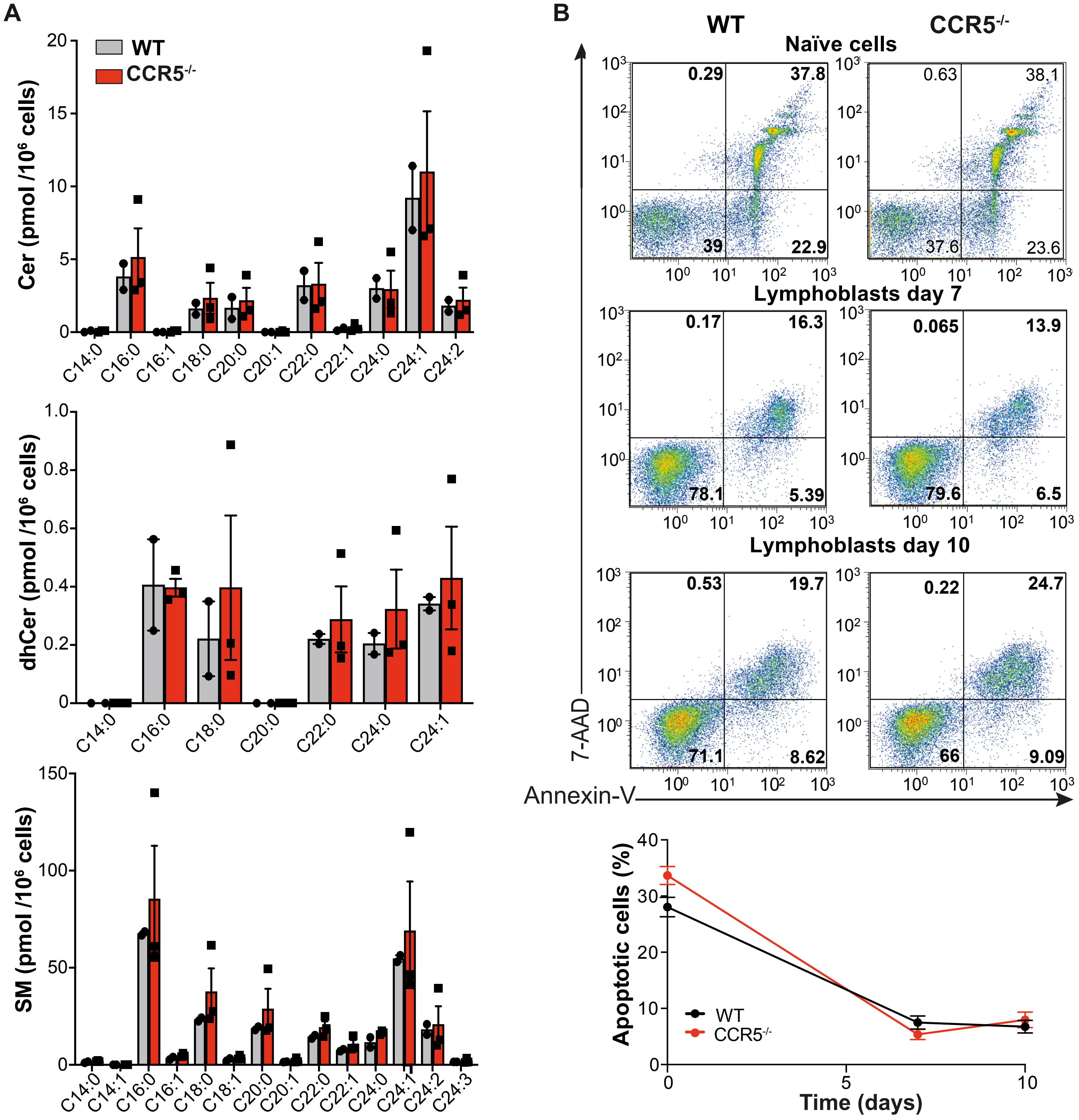

Appendix Figure S5. Analysis of sphingolipids and apoptosis in WT and CCR5$^{-/-}$ naïve OT-II cells. A. Cer (top), dhCer (center) and SM (bottom) levels in WT and CCR5$^{-/-}$ OT-II naïve cells. Values were normalized to the C17 standards and to cell number in each sample (n = 4). No significant differences were found between genotypes in any lipid species (two-tailed unpaired Student's t-test). **B.** Representative dot plots of WT and CCR5$^{-/-}$ OT-II naïve cells and IL-2 lymphoblasts at days 7 and 10, stained for the apoptosis markers 7-aminoactinomycin D (7-AAD) and annexin-V. Numbers represent the percentage of cells. The graph shows the quantification of doubled-stained cells in different experiments (bottom). Data are mean ± SEM (n = 4). No differences were found between genotypes (two-tailed unpaired Student's t-test).

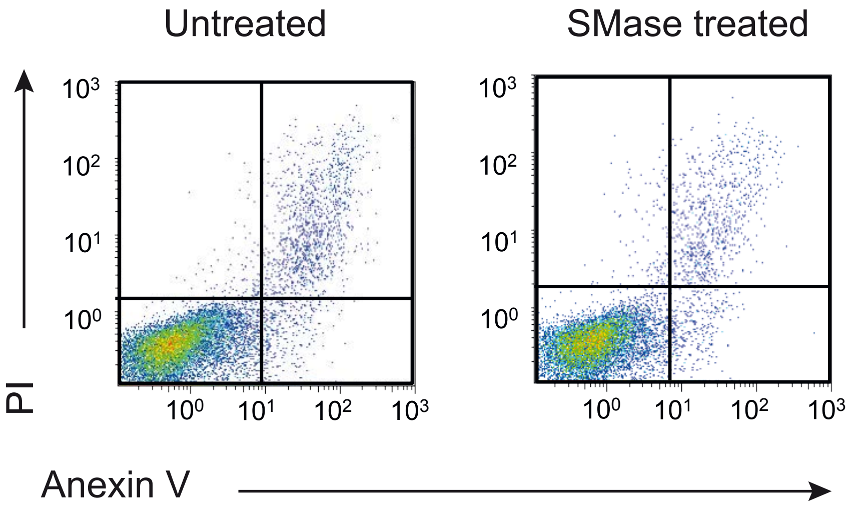

Appendix Figure S6. SMase treatment does not trigger apoptosis in lympholasts. Day 10 OT-II lymphoblasts were treated with SMase (0.5 U/ml, 1 h, 37ºC) and apoptosis determined by FACS analysis after staining with annexin-V and propidium iodide (PI). Plots shown for untreated (left) and SMase-treated cells (right) in a representative experiment (*n* = 4).

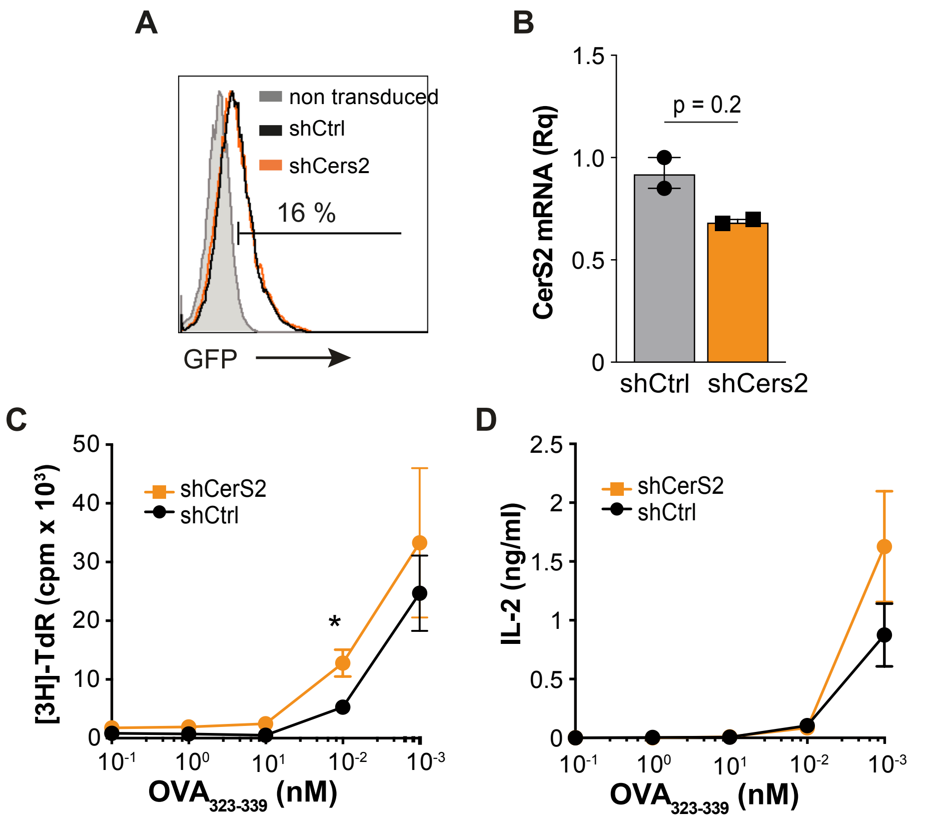

Appendix Figure S7. CerS2 silencing in primary CD4$^+$ lymphoblasts. A. Histograms showing GFP staining in shCtrl- (black line) and shCerS2-transduced OT-II CCR5$^{-/-}$ lymphoblasts (orange). Data for cells stained at 72 h post-transduction in a representative experiment. Gray area represents staining of non-transduced cells (negative control). The percentage of GFP$^+$ cells is indicated. **B.** Relative CerS2 mRNA levels in cells as in *A*. **C, D.** Determination of thymidine incorporation into DNA (*C*) and IL-2 levels (*D*) in the supernatant of shCtrl- (black line) and shCerS2-transduced (orange line) OT-II CCR5$^{-/-}$ lymphoblasts restimulated for 48 h with $OVA_{323\text{-}339}$ at the indicated concentrations. For *B-D*, data shown as mean ± SEM (n = 3). * $p < 0.05$, two-tailed unpaired Student's *t*-test.

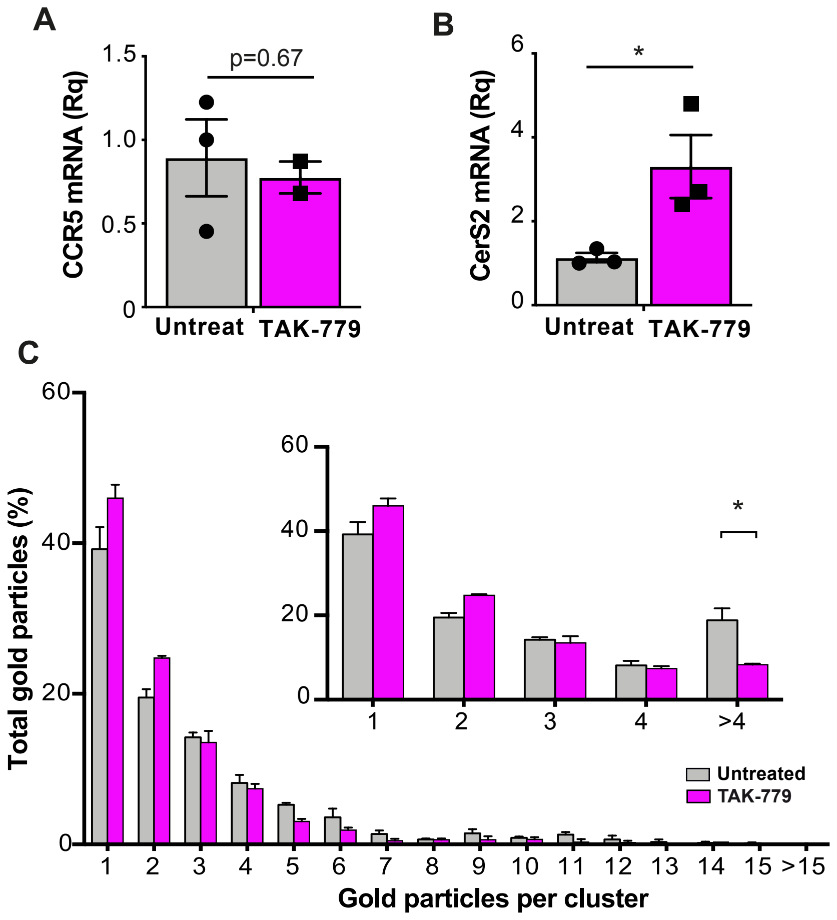

Appendix Figure S8. CCR5 blockade also impairs TCR nanoclustering in the CD4⁺ T cell hybridoma 2B4. A, B. Relative mRNA levels of CCR5 (*A*) and CerS2 (*B*) in CD3ε-activated 2B4 cells untreated (gray bars) or TAK-779-treated (10 μM; pink). **C.** Quantification (mean ± SEM) of gold particles in clusters of the indicated size in cell surface replicas of anti-CD3ε-labeled untreated (gray bars; *n* = 5 cells, 13266 particles) and TAK-779-treated 2B4 cells (black; *n* = 6 cells, 17654 particles). Insets show the distribution between clusters of one, two, three, four or more than four particles, and statistical analysis. * $p < 0.05$, one-tailed unpaired Student's *t*-test.

Appendix Table S1. Number of TCR nanoclusters/cell in the different experiments

Experiment	Cell type	Genotype or treatment	TCR nanoclusters/cell	*n*
Fig. 3A	Naïve OT-II cells	WT	68.3 ± 13.25	6
		CCR5$^{-/-}$	54 ± 6.7	6
Fig. 3B	IL-2-expanded OT-II lymphoblasts	WT	379.9 ± 49.1	8
		CCR5$^{-/-}$	163.3 ± 36	6
Fig. 3C	IL-15-expanded OT-II lymphoblasts	WT	600.9 ± 47.3	8
		CCR5$^{-/-}$	364 ± 53.6	7
Fig. 3G	Purified memory CD4^{+} T cells	WT	494.5 ± 84.9	5
		CCR5$^{-/-}$	351.4 ± 47.3	7
Fig. 4A	IL-2-expanded OT-II lymphoblasts	Medium	323.1 ± 58.5	5
		TAK-779	142.5 ± 22.3	6
Fig. 4B	IL-2-expanded OT-II lymphoblasts	Medium	211.5 ± 53.1	5
		TAK-779	195,6 ± 20,3	6
Fig. 4C	IL-2-expanded OT-II lymphoblasts	Vehicle	309.6 ± 65.9	6
		AMD3100	378.7 ± 74.5	7
Fig. 6E	IL-2-expanded OT-II lymphoblasts	Untreated	234.1 ± 58.1	5
		SMase-treated	208.5 ± 19.1	6
Fig. 6I	2B4 transduced cells	shCtrl	90.2 ± 16.5	6
		shCerS2	216 ± 63.7	7
Fig. 7A	Human CD4^{+} T cell lymphoblasts	WT	539.9 ± 80.4	5
		ccr5Δ32	337.7 ± 172	4

Nanoclusters are defined as aggregates of gold particles >2. *n*, number of cells analyzed. Data are mean ± SEM.

Appendix Table S2. RRID accession number and catalog reference of the reagents

REAGENT	SOURCE	IDENTIFIER
**Antibodies**		
Anti-mouse Vα_2TCR-PE (B20.1)	BD-Biosciences	Cat# 553289, RRID:AB 394760
Anti-mouse CD25-PE (PC61)	BD-Biosciences	Cat# 553866, RRID:AB 395101
Anti-mouse CD45.2-FITC (104)	BD-Biosciences	Cat# 561874,RRID:AB 10894189
Anti-mouse CD62L-FITC (MEL-14)	BD-Biosciences	Cat# 553150,RRID:AB 394665
Anti-mouse CD62L-APC (MEL-14)	BD-Biosciences	Cat# 553152,RRID:AB 398533
Anti-mouse CD69-PeCy7 (H1.2F3)	BD-Biosciences	Cat# 552879,RRID:AB 394508
Anti-mouse CXCR5 biotinylated (2G8)	BD-Biosciences	Cat# 551960,RRID:AB 394301
Anti-mouse biotin CD3ε (145-2C11)	BD-Biosciences	Cat# 553239,RRID:AB 394728
Anti-mouse unlabeled CD3ε (145-2C11)	BD-Biosciences	Cat# 553057,RRID:AB_394590
Anti-human biotin CD3 (OKT3)	Thermo Fisher Scientific	Cat# 13-0037-80, RRID:AB_1234956
Anti-mouse CD4-PeCy7 (RM4.5)	Thermo Fisher Scientific	Cat# 25-0042-81, RRID:AB_469577
Anti-mouse CD4-efluor450 (RM4.5)	Thermo Fisher Scientific	Cat# 48-0042-80, RRID:AB_1272231
Anti-mouse CD4-PacificBlue (RM4.5)	Thermo Fisher Scientific	Cat# MCD0428, RRID:AB_10372505
Anti-mouse IFNγ-APC (XMG1.2)	Thermo Fisher Scientific	Cat# 17-7311-82, RRID:AB_469504
Anti-mouse PD1-efluor780 (J43)	Thermo Fisher Scientific	Cat# 47-9985-82, RRID:AB_2574002
Anti-mouse phospho-GATA1-pSer142	Thermo Fisher Scientific	Cat# PA5-37581, RRID:AB_2554189
Anti-mouse CD44-Pe/Cy5 (IM7)	BioLegend	Cat# 103009, RRID:AB_312960
Anti-mouse CD44-APC (IM7)	BioLegend	Cat# 103011, RRID:AB_312962
Anti-mouse CerS2 (1A6)	Novus Biologicals	Cat# H00029956-M01A, RRID:AB_2132954
Anti-mouse CerS3 (6C12)	Novus Biologicals	Cat# H00204219-M02
Anti-mouse CerS6	Novus Biologicals	Cat# H00253782-M01, RRID:AB_2133107
Anti-mouse CerS4 (polyclonal)	Sigma-Aldrich	Cat# SAB4503164, RRID:AB_10746317
Anti-mouse CD3ζ (449)	Purified from hybridoma, this study	N/A
Anti-mouse β-actin (AC-15)	Sigma-Aldrich	Cat# A1978, RRID:AB_476692
Anti-mouse GATA1 (ab11852)	Abcam	Cat# ab11852, RRID:AB_298635
Anti-acetyl histone H3Lys9 (CS200583)	EMD-Millipore	Cat# 07-352, RRID:AB_310544
purified IgG rabbit (PP64)	EMD-Millipore	Cat# PP64, RRID:AB_97852
**Virus strains**		
rVACV-OVA virus	a gift of J.W.Yewdell; NIH, Bethesda, MD	N/A
**Biological Samples**		
Healthy adult blood samples	Fundació ACE (Barcelona, Spain)	NA
**Chemicals, peptides, recombinant proteins**		
OVA(323–339) peptide	CNB Peptide facility	N/A
Sphingomyelinase (Bacillus cereus)	Sigma-Aldrich	Cat# S7651
TAK-779	Sigma-Aldrich	Cat# SML0911
AMD-3100	Sigma-Aldrich	Cat# 239820
poly-L-lysine	Sigma-Aldrich	Cat# P4707

Ceramide (bovine spinal cord)	Sigma-Aldrich	Cat# 22244
Protein A-Gold	Sigma Aldrich	Cat# P6730
Streptavidin-agarose	Sigma Aldrich	Cat# S1638
Ceramide from bovine spinal cord	Sigma Aldrich	Cat# 22244
NIP-OVA	Biosearch Technologies	Cat# N-5041
NIP-KLH	Biosearch Technologies	Cat# N-5042
Aluminium hydroxide gel	InvivoGen	Cat# vac-alu-250
Streptavidin APC	Thermo Fisher Scientific	Cat# 17-4317-82
Streptavidin PerCP-Cy5	Thermo Fisher Scientific	Cat# 45-4317-82; RRID: AB_10311495
Dynabeads™ M-450 Tosylactivated	Thermo Fisher Scientific	Cat# 14013
Proteinase K	Thermo Fisher Scientific	Cat# 25530049
Streptavidin BV786	BD Bioscience	Cat# 563858
Recombinant murine CCL4	PeproTech	Cat# 250-32
Recombinant murine IL-2	PeproTech	Cat# 212-12
Recombinant murine IL-15	PeproTech	Cat# 210-15
Recombinant human IL-2	PeproTech	Cat# AF-200-02
LIVE/DEAD™ Fixable Near-IR Dead Cell Stain Kit	Molecular Probes	Cat# 10154363
DAPI Fluoromount-G	Southern Biotech	Cat# 0100-20
Thymidine, [Methyl-3H]	Pelkin Elmer	Cat# NET027W001MC
SureBeads™ Protein G Magnetic Beads	Bio Rad	Cat# 1614023
LipoD293™ In Vitro DNA Transfection Reagent	SignaGen Laboratories	Cat# SL100668
Cholesterol	Avanti Polar Lipids	Cat#700100
Soybean L-α-phosphatidylcholine	Avanti Polar Lipids	Cat#840054C
Egg Sphingomyelin	Avanti Polar Lipids	Cat#860061C
C12 Ceramide (d18:1/12:0)	Avanti Polar Lipids	Cat# 860512
C16 Ceramide (d18:1/16:0)	Avanti Polar Lipids	Cat# 860516
C18 Ceramide (d18:1/18:0)	Avanti Polar Lipids	Cat# 860518
C24 Ceramide (d18:1/24:0)	Avanti Polar Lipids	Cat# 860524
C24:1 Ceramide (d18:1/24:1(15Z))	Avanti Polar Lipids	Cat# 860525
C16 Dihydroceramide (d18:0/16:0)	Avanti Polar Lipids	Cat# 860634
C18 Dihydroceramide (d18:0/18:0)	Avanti Polar Lipids	Cat# 860627
C24 Dihydroceramide (d18:0/24:0)	Avanti Polar Lipids	Cat# 860628
C24:1 Dihydroceramide (d18:0/24:1(15Z))	Avanti Polar Lipids	Cat# 860629
12:0 SM (d18:1/12:0)	Avanti Polar Lipids	Cat# 860583
16:0 SM (d18:1/16:0)	Avanti Polar Lipids	Cat# 860584
18:0 SM (d18:1/18:0)	Avanti Polar Lipids	Cat# 860586
24:0 SM	Avanti Polar Lipids	Cat# 860592
24:1 SM	Avanti Polar Lipids	Cat# 860593
C12 Glucosyl(β) Ceramide (d18:1/12:0)	Avanti Polar Lipids	Cat# 860543
C17 sphinganine (d17:0)	Avanti Polar Lipids	Cat# 860654
C17 sphinganine-1-phosphate (d17:0)	Avanti Polar Lipids	Cat# 860655
Chloroform	JT Baker	Cat# 15588534
Water for LC-MS	JT Baker	Cat# 15568664
Methanol for LC-MS	Fisher Chemical	Cat# 15611630
Ammonium formate for LC-MS	Fisher Chemical	Cat# 11377490
Formic Acid for LC-MS	Fluka	Cat# 15671400
**Critical commercial assays**		
ELISA MAX™ Deluxe Set Mouse IL-2	BioLegend	Cat# 431004

Mouse Memory T cell CD4+/CD62L-/CD44hi Column Kit	R&D Systems	Cat# MCD45
Dynabeads™ Untouched™ Mouse CD4 Cells Kit	ThermoFisher	Cat# 11415D
Amplex™ Red Cholesterol Assay Kit	ThermoFisher	Cat# A12216
EasySep™ Human CD4+ T Cell Enrichment Kit	StemCell	Cat# 19052
SBA Clonotyping System-HRP	SouthernBiotech	Cat# 5300-05
RNeasy Mini Kit	QIAGEN	Cat# 74104
EZ-ChIP™	Millipore	Cat# 17-371
**Experimental models: Cell lines**		
HEK-293 T	ATCC	Cat# CRL-3216, RRID:CVCL_0063
2B4 T cell hybridoma	J. Ashwell, Bethesda	RRID:CVCL_4Z38
M.mζ-SBP	(Swamy and Schamel, 2009)	N/A
**Experimental models: Organisms**		
B6.129P2-Ccr5tm1Kuz (CCR5$^{-/-}$)	The Jackson Laboratory	Cat# JAX:005427, RRID:IMSR_JAX:005427
C57BL/6J	The Jackson Laboratory	Cat# JAX:000664, RRID:IMSR_JAX:000664
B6.Cg-Tg(TcraTcrb)425Cbn/J (OT-II)	The Jackson Laboratory	Cat# JAX:004194, RRID:IMSR_JAX:004194)
OT-II-CCR5$^{-/-}$	(González-Martín et al., 2011)	N/A
B6.SJL-Ptprca Pepcb/Boy	The Jackson Laboratory	Cat# JAX:002014, RRID:IMSR_JAX:002014
CD3ε$^{-/-}$	(DeJarnette et al., 1998)	N/A
**Oligonucleotides**		
Primers for qRT-PCR, see Table S1		N/A
**Software and Algorithms**		
Bayesian JAGS code		Provided as supplementary material
FlowJo v10	Tree Star	RRID:SCR_008520
GraphPad Prism v6 & v7	GraphPad	RRID:SCR_002798
NIH Image J	NIH Image	RRID:SCR_003073
UCSC Genome browser	University of California Santa Cruz	RRID:SCR_005780
GTRD v17.04	Gene Transcription Regulation Database	http://gtrd17-04.biouml.org/
Venny 2.1	BioinfoGP, CNB/CSIC	RRID:SCR_016561
Adobe Photoshop CS5	Adobe Software	RRID:SCR_014199
Adobe Illustrator CS5	Adobe Software	RRID:SCR_010279
**Others**		
Brefeldin A	Sigma-Aldrich	Cat# B7651
IntraPep Permeabilization reagent	Beckman Coulter	Cat# A07803
GIPZ lentiviral mouse CerS2 shRNA (clones V3LMM_454307, V3LMM_454309, V3LMM_454311)	Dharmacon	Cat# RMM4532-EG76893
Bio-Beads™ SM-2 Resin	BioRad	Cat# 1523920
Acquity C8 UPLC column	Waters	Cat# 186002878

Appendix Table S3. List of primers used for RT-qPCR analyses

Gene Symbol	Primer (5' – 3') Forward	Reverse
mCerS2	GGCGCTAGAAGTGGGAAAC	TCGAATGACGAGAAAGAGCA
mCerS3	GCTACACCTCTAGCAAATGCAC	ATCTTTCAACCTGGCGCTCT
mCerS4	AGATAAAGCCCAACCCGGTG	GTCTCCTGAACCAGCGTTGA
mCerS5	CCAATGCTGGTTTCGCCATC	AGAACCAAGGCATCGACCAG
mCerS6	GGAGCTGTCATTTTATTGGTCTTT	GGAACATAATGCCGAAGTCC
mASAH1	TGAAGATGGTGGATCAAAAGC	ACATCTGCAATTCCCCTCA
mACER2	GTGTGGCATATTCTCATCTG	TAAGGGACACCAATAAAAGC
mACER3	TGACCTTGTTCGTCGCTGAG	AGCAATGTACCGCTTCTCCA
mSMPD1	TGGTTCTGGCTCTGTTTGACTCCA	TCAGCTGATCTTGGCGAGACTGTT
mSMPD2	GGTGCTCAACGCCTATGTG	CGTCTGCCTTCTTGGATGTG
mSMPD3	AGAAACCCGGTCCTCGTACT	CCTGACCAGTGCCATTCTTT
mSMPD4	GCCAACGACCTGGACGAGATC	GCGAGTGTGAACTGCCTGAG
CCR5	TCCGTTCCCCCTACAAGAGA	TTGGCAGGGTGCTGACATAC
mTcra	CCAGACTGGCAGCAAGAAGAAAAT	TCACAGCTCCCCACCATATTC
mTcrb	GTGCTGTGAAGGATGGCAACT	CGGCAGGGTCAGGGTTCT
mCD3d	TGTGCAAGTCCATTACCGAAT	AAAGCAGTAGACGCCCAAAG
mCD3g	GAGAAGCAAAGAGACTGACATGG	TTATTTGTCTGGGCTACAGTGC
mCD3e	AACACTTTCTGGGGCATCCT	ATGTTCTCGGCATCGTCCT
mCD3z	GCACGATGGCCTTTACCA	CAAGTGACATCAGCAGGTGAA
mGATA-1	TGCTCTCTTCTTGAGGCATAGATT	CCAGCCCTGCTGTTTAGAGTC
mHISTH3	GCTAAGCTTAACTCTCCCGGT	AAGCGCCCAGCAGCC
hCerS2	GACGGAGTACACGGAGCAG	CGTTCCCACCAGAAGTAATCA
hCerS4	TGGTGCTGCTGTTACACGAT	TGATACTGCATGTAGTTGACCATC
hCerS5	CACATCCTCTCGGTGTTCC	CAGGGTTTGGCAATAAATCG
hCerS6	CGACTGGGTATATTTCCTCTCTG	GGAAGGGTAAGGTCCAACG
hASAH1	CACGCTGATTGGGTGTGTA	CGATGTTCAGCTTGTATTTCTTGA
hACER2	TGTGGTTCCCCAGAAGGTAT	ACGTCGTAACCGCAGACAG
hACER3	CCTGAGATATAGGCCAAAAGTGA	GCCAGGTCTATGGTAGGTGCT
hSMPD1	TGGCTCTATGAAGCGATGG	TGGGGAAAGAGCATAGAACC
hSMPD3	TGGTACCCAAGAACTGCTACG	AAAACCCAGAAACTGCCTTG
18S rRNA	GAGAAACGGCTACCACATCC	GGGTCGGGAGTGGGTAAT

Bayesian code for the R-language

```
# require(rjags)
# The array "p" contains 4 different cluster size counts from different
experiments for the same mouse and time
cluster.jags.multix4 <- function(p) {
  p1 <- p[1,]
  p2 <- p[2,]
  p3 <- p[3,]
  p4 <- p[4,]
  data <-
list(p1=p1,p2=p2,p3=p3,p4=p4,m=length(p1),N1=sum(p1),N2=sum(p2),N3=sum(p3),N4=su
m(p4))

  modelstring="
  model {
  for(n in 1:(m-1)) {
     pi1[n] <- b1^(n-1)*(1-b1) # Analytical distribution described in the main
text
     pi2[n] <- b2^(n-1)*(1-b2)
     pi3[n] <- b3^(n-1)*(1-b3)
     pi4[n] <- b4^(n-1)*(1-b4)
  }
  pi1[m] <- b1^(m-1)  # Analytical distribution described in the main text
  pi2[m] <- b2^(m-1)
  pi3[m] <- b3^(m-1)
  pi4[m] <- b4^(m-1)

  p1 ~ dmulti(pi1,N1) # The counts are given by a multinomial distribution with
probabilities "pi"
  p2 ~ dmulti(pi2,N2)
  p3 ~ dmulti(pi3,N3)
  p4 ~ dmulti(pi4,N4)

  b1 ~ dbeta(A,B) # Priors for the parameters b1.
  b2 ~ dbeta(A,B)
  b3 ~ dbeta(A,B)
  b4 ~ dbeta(A,B)
  A ~ dunif(0,1000) # Hyperpriors for A and B (0 = uniform distribution,
Infinity=peaked distribution)
  B ~ dunif(0,1000)
  }"

  model=jags.model(textConnection(modelstring), data=data,n.chains = 3) # Create
jags model
  update(model,n.iter=10000) # Burning phase of the MCMC model
  output=coda.samples(model=model,variable.names=c("A","B","b1","b2","b3","b4"),
n.iter=15000, thin=1) # Sample data
  print(summary(output)) # Print estimated parameters
  return(output) # return matrix of results for post-processing
}
```
