# Supplementary material for: CCR5 deficiency impairs CD4+ T‐cell memory responses and antigenic sensitivity through increased ceramide synthesis
Source: EMBO J. 2020 Jun 11;39(15):e104749. doi: 10.15252/embj.2020104749 (PMC7396835; doi:10.15252/embj.2020104749)
Supplement: Supplementary file 2 — Expanded View Figures PDF [file EMBJ-39-e104749-s002.pdf]

Expanded View Figures

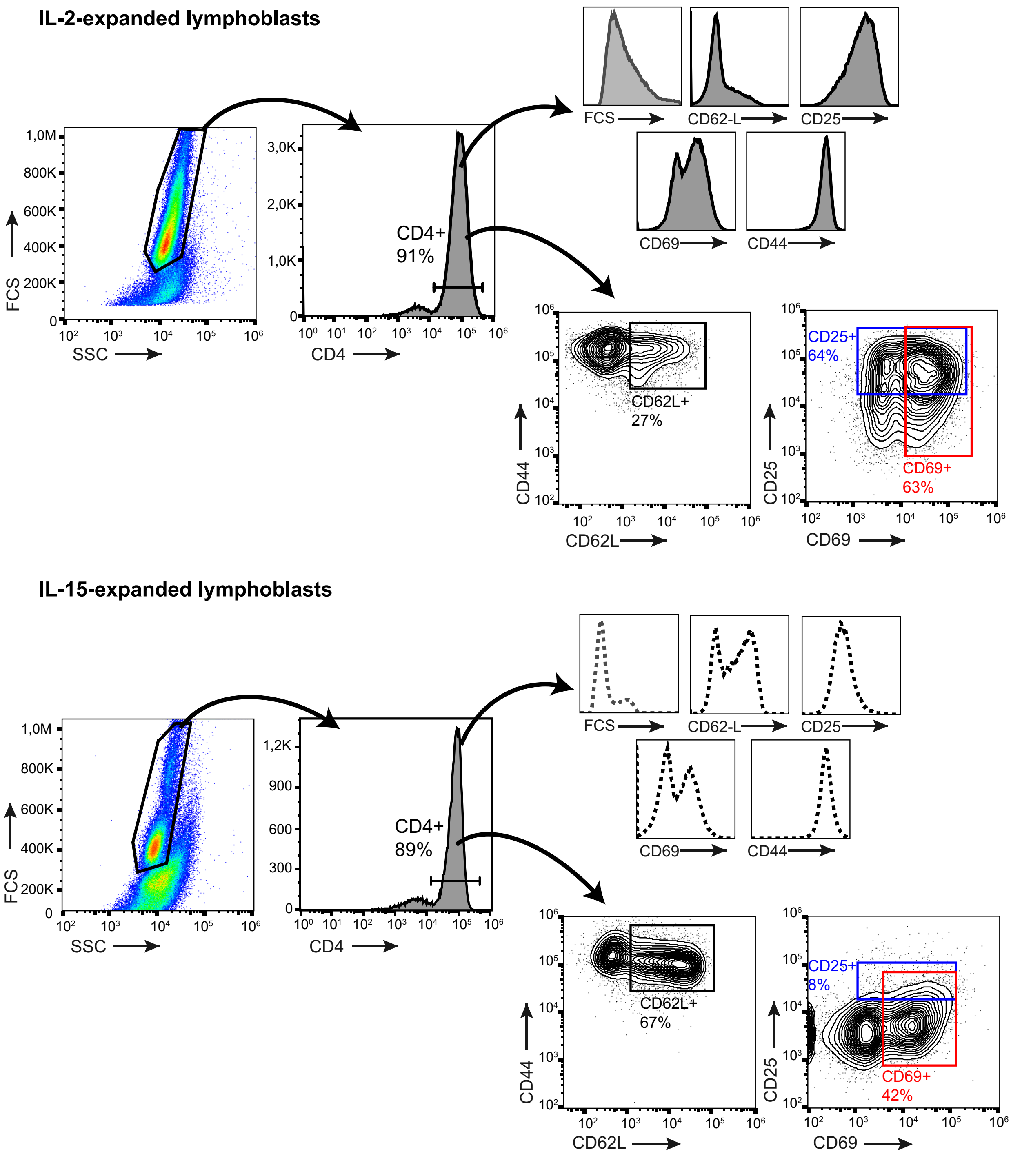

Figure EV1. Gating strategy for characterization of IL-2 and IL-15-expanded OT-II lymphoblast.

Representative examples of the gating strategy used to characterize the IL-2- and IL-15-expanded OT-II lymphoblasts using the indicated markers.

 © 2020 The Authors

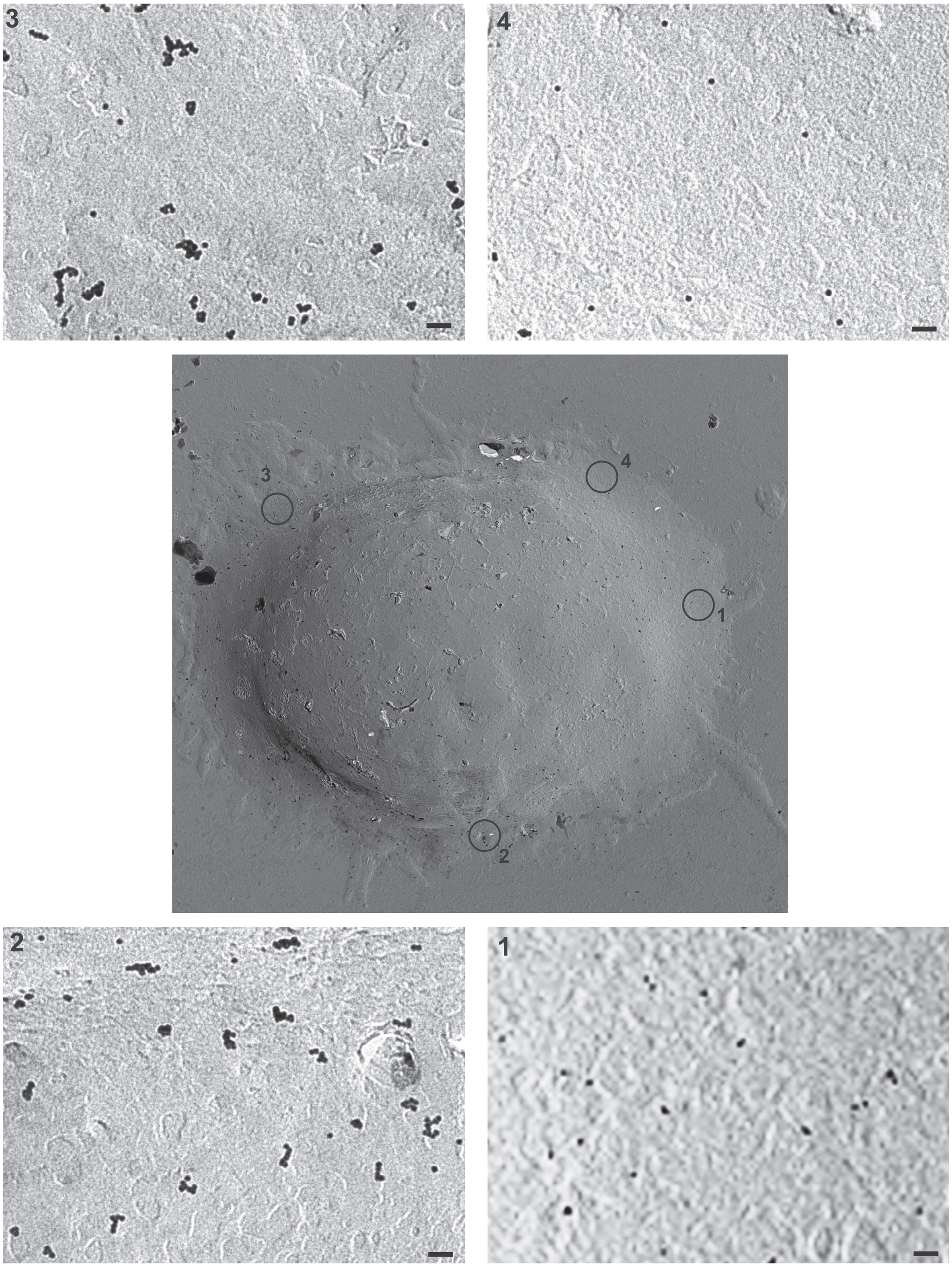

Figure EV2. Analysis of TCR molecules by electron microscopy.

Representative cell surface replica of a IL-15-expanded WT lymphoblast stained with anti-CD3ε antibody and gold-conjugated protein A. Some cell areas have been enlarged to show the distribution of the TCR-stained molecules. Scale bar, 50 nm.

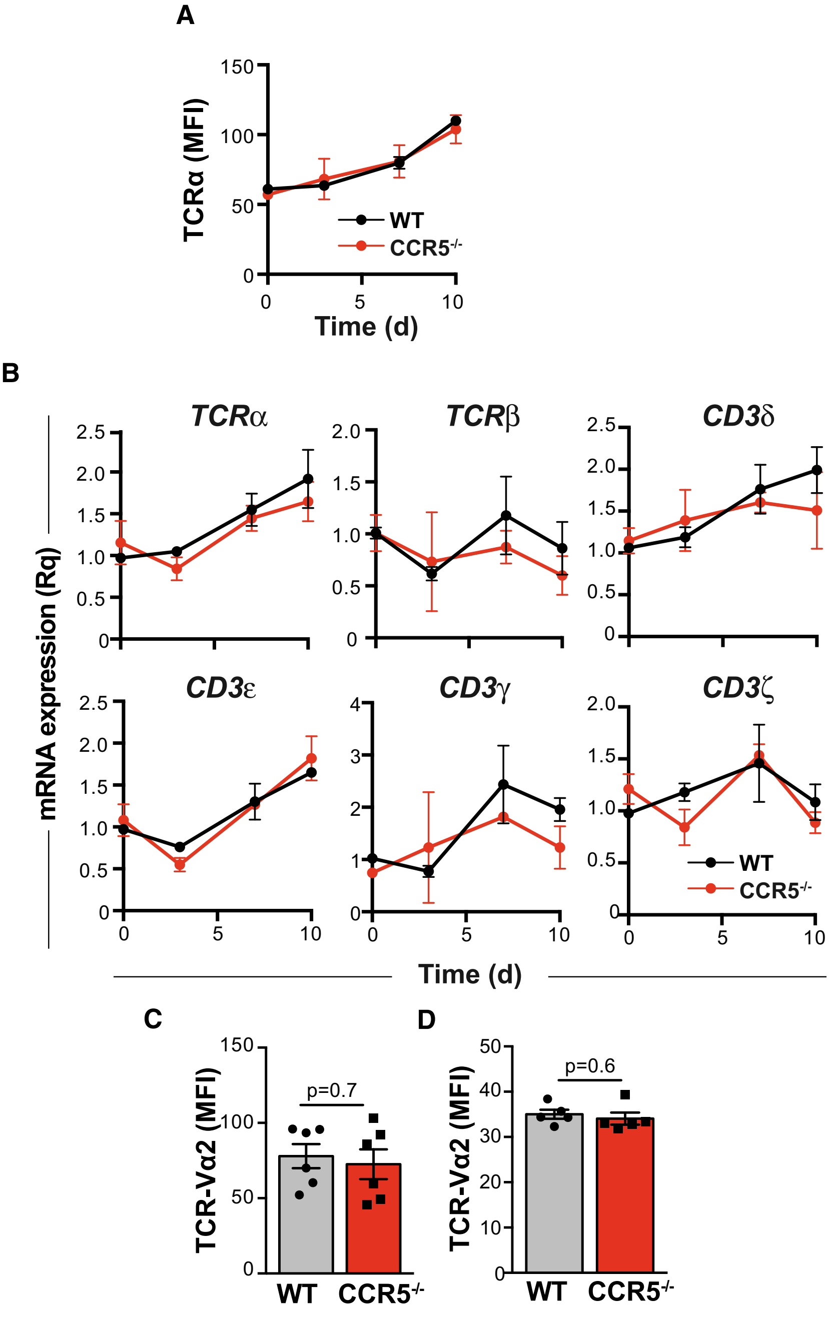

Figure EV3. CCR5 does not affect TCR expression.

A Quantification of mean fluorescence intensity of TCRα (Vα2) surface staining in IL-2-expanded, $OVA_{323-339}$-activated WT and $CCR5^{-/-}$ OT-II cells on the days indicated.

B Relative mRNA levels for the indicated TCR chains in cells as above.

C Mean fluorescence intensity of TCRα (Vα2) surface staining in IL-15-expanded, $OVA_{323-339}$-activated WT and $CCR5^{-/-}$ OT-II cells.

D Mean fluorescence intensity of TCRα (Vα2) surface staining in $CD45.2^{+}/CD4^{+}$ memory cells isolated from NIP-OVA-immunized WT and $CCR5^{-/-}$ mice.

Data information: In all cases, data are presented as mean ± SEM ($n \geq 3$). Differences were not significant using a two-way ANOVA with Bonferroni *post-hoc* test (A, B) or two-tailed Student's *t*-test (C, D).

 © 2020 The Authors

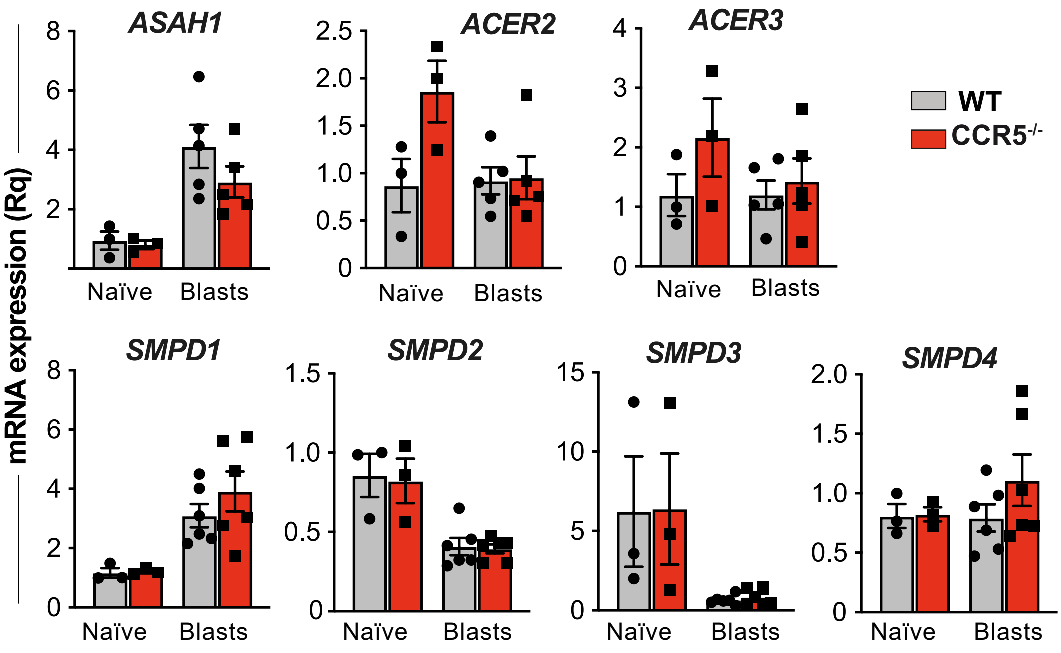

Figure EV4. Characterization of enzymes involved in ceramide metabolism.

Relative mRNA levels for acid ceramidase (ASAH1), alkaline ceramidases 2 and 3 (ACER2, ACER3), acid sphingomyelinase (SMPD1), and neutral sphingomyelinase (SMPD)-2, neutral sphingomyelinase-3, and neutral sphingomyelinase-4 in WT and CCR5$^{-/-}$ OT-II naïve cells and lymphoblasts (day 10).
Each data point is the average of triplicates in an independent experiment (n = 3 or 5). The mean ± SEM is also provided. There were no significant differences between genotypes at any given time point (two-tailed unpaired Student's t-test).

© 2020 The Authors

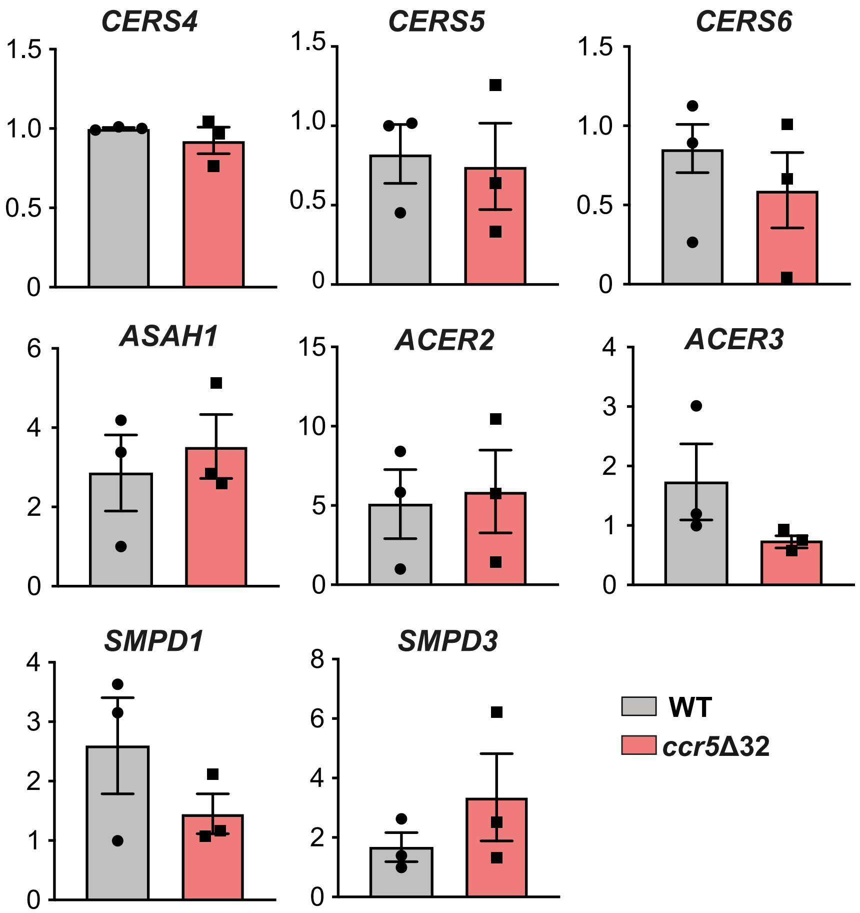

Figure EV5. Characterization of enzymes involved in ceramide metabolism in human CD4^{+} T cells.

Relative mRNA levels for CERS4, CERS5, CERS6, ASAH1, ACER2, ACER3, acid SMase (SMPD1), and neutral SMase (SMPD3) in primary lymphoblasts from healthy donors homozygous for *ccr5*Δ32 (red bars) or who do not bear this polymorphism (WT; gray). Each data point is the average of triplicates from a donor (n = 3). Bars represent the mean ± SEM. There were no significant differences between genotypes for these enzymes (two-tailed unpaired Student's t-test).

© 2020 The Authors
